# The Effects of Anesthetics on the Cortex—Lessons From Event-Related Potentials

**DOI:** 10.3389/fnsys.2020.00002

**Published:** 2020-02-11

**Authors:** Dana Baron Shahaf, Gregory M. T. Hare, Goded Shahaf

**Affiliations:** ^1^Rambam Health Care Campus, Haifa, Israel; ^2^Department of Anesthesia, St. Michael’s Hospital, University of Toronto, Toronto, ON, Canada; ^3^St. Michael’s Hospital Center of Excellence for Patient Blood Management, St. Michael’s Hospital, Toronto, ON, Canada; ^4^Department of Physiology, University of Toronto, Toronto, ON, Canada; ^5^Keenan Research Centre for Biomedical Research, in the Li Ka Shing Knowledge Institute, Toronto, ON, Canada; ^6^NeuroIndex LTD. Beit Tavor, Yokneam, Israel

**Keywords:** anesthesia, event-related potentials (ERP), cortex, EEG, attention, perception

## Abstract

Consciousness while under general anesthesia is a dreadful condition. Various electroencephalogram (EEG)-based technologies have been developed, on the basis of empirical evidence, in order to identify this condition. However, certain electrophysiological phenomena, which seem strongly related with depth of anesthesia in some drugs, appear less consistent with those of other anesthetic drugs. There is a gap between the complexity of the phenomenon of consciousness and its behavioral manifestations, on the one hand, and the empirical nature of the reported electrophysiological markers, which are associated with it, on the other hand. In fact, such a gap might prevent us from progressing toward unified electrophysiological markers of consciousness while under anesthesia, which are applicable to all anesthetic drugs. We believe that there is a need to bridge this conceptual gap. Therefore, in this work, we will try to present a theoretical framework for such bridging. First, we suggest focusing on neuropsychological processes, which seem to have a clear role in the behavioral manifestations of consciousness while under anesthesia but seem, nevertheless, better defined than consciousness itself—such as perception and attention. Then, we suggest analyzing the effects of anesthesia upon these neuropsychological processes, as they are manifested in the EEG signal. Specifically, we will focus on the effects of anesthesia on event-related potentials (ERPs), which seem more easily associable with neuropsychological modeling.

## The Problem

### The Practical and Theoretical Challenges

Consciousness while under general anesthesia is a dreadful condition (Osterman et al., 2001). Various electroencephalogram (EEG) markers were developed in order to identify this condition in real time (Bruhn et al., 2006). Specifically, it seems that three major types of overt electrophysiological markers are at the focus of EEG monitoring of consciousness while under anesthesia. Owing to practical considerations, and especially the ability to rapidly place electrodes below the hairline, it seems that the most prevailing types of markers are based on: (i) the relation between the frontal activity in higher-frequency bands and the activity in lower-frequency bands, which is expected to increase between unconsciousness and consciousness (Morimoto et al., 2004); and (ii) the degree of irregularity of the frontal activity, which is also expected to increase between unconsciousness and consciousness (Vakkuri et al., 2004). The third type of electrophysiological markers, which seems to be strongly associated with the change between consciousness and unconsciousness, is anteriorization of certain types of activity, mainly of activity in the alpha frequency band (~8–13 Hz). Thus, we see, in association with loss of consciousness, an increase in the ratio between activity in anterior electrodes and activity in posterior electrodes (Purdon et al., 2013). However, this type of markers requires also posterior electrodes, which are above the hairline and are usually more cumbersome to place effectively.

The precision of all these marker types was challenged significantly over the last few years in multiple publications (Schuller et al., 2015; Gaskell et al., 2017), including guidelines to limit the use of markers (e.g., Pandit and Cook, 2013). Owing to the clinical use of such markers, these publications have significant practical implications and are not merely of theoretical interest. Some of the criticism relates to the potential impact of electromyographic (EMG) activity from the forehead and ocular muscles, especially for the markers that are measured from anterior electrodes. On the one hand, movement and EMG might be related with awareness; the concern is for conditions in which muscle relaxants reduce EMG signal and thereby the markers when the patient might be awake and be aware owing to ineffective anesthesia (Schuller et al., 2015).

Another challenge relates to the fact that all of these markers seem to be well evident in certain anesthetic drugs yet much less evident in other drugs, which may relate with other EEG patterns. Furthermore, more complicated EEG patterns may relate with specific drug combinations (Hirota et al., 1999; Hans et al., 2004).

The impact of the EMG on the electrophysiological markers seems to be a major concern that needs to be addressed. However, the fact that electrophysiological markers, which seem strongly associated with consciousness and its reduction by certain anesthetic drugs, do not demonstrate a similar association with other drugs is theoretically challenging. Consciousness while under anesthesia would have been expected to have a robust impact on cortical activity (Mashour, 2006) and EEG. The lack of such a comprehensive EEG pattern (Harrison and Connolly, 2013), which could be found for all anesthetic drugs that induce loss of consciousness, is puzzling. Attempts at explaining these electrophysiological inconsistencies seem to focus on the molecular and biochemical differences between the drug groups (Sou et al., 2006), as well as on some related variability in the impact of differing drugs on certain brain regions (Mashour and Avidan, 2017). For sure, in principle, molecular and cellular processes impact EEG. However, we are looking for a robust EEG marker for a neuropsychological process (consciousness and its reduction under anesthesia), which is actually shared by all the various drugs. Intuitively, such a marker should exist; however, we seem to encounter a theoretical barrier in this regard—we do not have a sufficiently clear operational definition of consciousness while under anesthesia and its reduction by the drugs (Block, 2001). On top of that, we do not have a clear association between such an operational definition of consciousness and its expected manifestation as a marker in the EEG activity.

The gap between consciousness while under anesthesia, its neuropsychological embodiment, its operational definition, and the possible manifestation of this operational definition in the EEG signal is the focus of this manuscript. We hope to suggest that such an operational definition of consciousness while under anesthesia, and its manifestation in the EEG signal, may lead to general markers for its identification, applicable to any type of anesthetic drug. In general, aiming at such a task for consciousness, on its elusive definition, would be highly challenging, and it should be emphasized that we only refer in this manuscript to the practical derivative of awareness of aversive and nociceptive conditions under anesthesia and during surgery.

### The Approach Presented in This Manuscript

Certain neuropsychological processes have been related to raw EEG markers (Loo and Barkley, 2005). However, it seems that deeper neuropsychological interpretation of the electrophysiological signal may often be given in the context of behavioral tasks, which are used for neuropsychological assessment, and when the signal analysis is done in relation to timed stimuli and responses during these tasks (Key et al., 2005). Regularly, the tasks that are used in this context are versions of standard neuropsychological tests that measure participant responses (e.g., correct and incorrect responses and response time). Therefore, the neuropsychological interpretation of the EEG event-related potentials (ERPs) seems more straightforward than raw EEG analysis, which lacks specific reference to the behavior of the participant at the time of measurement.

Notably, despite this potential “neuropsychological appeal” of ERP, the major markers for monitoring consciousness while under anesthesia are based on raw EEG. Thus, despite significant research (which will be discussed in some detail below), utilization of the ERP analysis in clinical monitoring of patients’ consciousness while under anesthesia is rather limited in comparison with raw EEG-based monitoring (Al-Kadi et al., 2013). However, in this work, we step back from implementation, for the sake of theoretical discussion of the major findings, which were, nevertheless, reached in multiple studies of the effects of anesthetic drugs on ERP. Clearly, while at the sub-anesthetic dose, ERP protocols can involve also the measurement of patient’s responses, at higher doses, which induce loss of consciousness; only protocols that do not require active participation are feasible (for example, auditory oddball protocols).

We will explore the relevant ERP–anesthetics literature from a very specific “prism,” which we use to analyze the ERP signal. As a first step, we present, in short, the basis for this “prism,” by which we relate to the ERP signal as a superposition of two types of processes, which could be summarized as perception-related processes and attention-related processes (Shahaf and Pratt, 2013; Shahaf et al., 2015). Thereafter, we use this prism as a guide for reviewing the relevant ERP–anesthetics literature. Note that this “prism” would mean we will try to substitute the somewhat evasive entity of consciousness while under anesthesia by the somewhat better-understood entities of perception- and attention-related processes—the rationale for this substitution will be discussed below.

Significant basic research has taken quite the opposite direction, namely, of distinguishing between consciousness and processes such as attention and perception (van Boxtel et al., 2010). However, this basic research focuses on consciousness to subtle types of stimuli, which are not evoking an attentive response. On the other hand, in this manuscript, we discuss consciousness, or awareness, of aversive and nociceptive stimuli during the operation, which in all likelihood would evoke perception and attention activities.

## Event-Related Potential as a Superposition of Two Types of Processes—Attention Related and Perception Related

### Event-Related Potential as a Superposition of Attention-Related and Perception-Related Processes

“*Clarification: In this section, we present a division of the ERP signal to underlying attention-related processes and perception-related processes. If this division might still seem hypothetical to the reader (despite the evidence provided in this section), it should be remembered that it is merely provided, in the current manuscript, to present a comprehensive theoretical framework. In fact, when we discuss the impact of anesthetics on ERP, in the sections below, we base the discussion on the standard literature of ERP waves and mainly on N1 and P3. This section enriches, in our view, the main message of this manuscript regarding the impact of anesthetics on the ERP signal. However, this main message could be derived even if the reader would find this section more hypothetical*.”

The analysis of the EEG signal in various experimental paradigms is, often, based on averaging the sampled activity for a period of time preceding and/or succeeding repetitive events, which may be stimuli or responses. Notably, the standard analysis of this averaged ERP is often based on the amplitude and/or latency of a limited set of waveforms, which stand out after, or before, the time-locked averaging (Picton et al., 2000). However, these standard waveforms are not always all that standard. In fact, various samples could be either inconclusive or with multiple peaks in the relevant timeframes, in which specific waveforms are defined (Kiesel et al., 2008). Furthermore, even when the waveforms are conspicuous, they often appear to reflect a superposition of underlying neurophysiological processes (Luck, 2004). Nevertheless, vast evidence has accumulated on underlying neurophysiological processes, for each of the standard waveforms and on their neuropsychological interpretation (Key et al., 2005). Still, it seems that significantly improved characterization of the underlying neurophysiological processes could be extracted from the signal, if it is analyzed in a subtler manner and not only by its overt and standard waveforms.

Various methods have been employed for extracting subtler information from the averaged ERP signal. Especially noteworthy and prevalent are various blind source separation methods, such as principal component analysis and independent component analysis (Onton and Makeig, 2006). These methods identify patterns that are not necessarily overt to begin with. However, the neurophysiological interpretation of these patterns is not always straightforward, as assumptions employed by these methods do not necessarily agree with neurophysiological understanding (Luck, 2004). Specifically, a most problematic set of assumptions, employed in one way or the other, by various blind source separation methods relates to the degree of independence of the underlying processes. It seems that assuming independence between various brain processes (e.g., between perception and attention) is somewhat doubtful considering our understanding of the brain’s function.

Altogether, it seems that prevailing averaged ERP analysis methods, whether based on the standard waveforms or on blind source separation, involve assumptions that may have only limited neurophysiological justification. Therefore, we developed the principles of a comprehensive method, which may minimize *a priori* assumptions regarding the expected nature of the processes, which underlie the ERP signal (Shahaf and Pratt, 2013; Shahaf et al., 2015). When we implemented the method, we found that the entire averaged ERP signal may be decomposed to two basic sets of processes (Shahaf et al., 2015): the first set of processes is related to early ERP activation and to standard ERP waves, which are traditionally related to the neuropsychological processes of sensation and perception (we will term this set below the “perception-related process”), and the second set of processes is related to lasting ERP activation and to standard ERP waves, which are traditionally related to the neuropsychological processes of attention and higher cognitive processing (we will term this set below the “attention-related process”). In further studies, we showed that it is possible to specify effective markers, for the attention-related process in the delta band activity from stimulus onset to about 600 ms after stimulus and for the perception-related process in the alpha band activity in the first 200 ms after stimulus onset (Shahaf et al., 2015). Specifically, with regard to the attention-related marker, we showed its efficacy in various clinical populations, such as patients with depression (Shahaf et al., 2017; Isserles et al., 2018), attention deficit hyperactivity disorder (ADHD) patients (Shahaf et al., 2018b), migraine patients (Shahaf et al., 2018a), and patients in rehabilitation after stroke (Bartur et al., 2017, 2019).

### Accordance With the Accepted Interpretation of Standard Event-Related Potential Waves

We found that our perception-related process associates with early ERP waves, which occur within 50–200 ms after stimulus onset, such as N1, and that our attention-related process associates with later ERP waves, which occur up to 600 ms after stimulus onset, such as P3 (Shahaf et al., 2015). The associations between the earlier waves (e.g., N1) with sensation and perception and of the later waves (e.g., P3) with higher cognitive processing, which involves attention, are generally accepted (Key et al., 2005). Maybe, if one looks for a specific and sensitive marker for the early process, which we termed “perception related,” or for the lasting process, which we termed “attention related” in a given protocol and clinical group, the standard waves will not be sufficiently precise, owing to the constraints described above. But for the sake of identifying tendencies at the group level, there is ample evidence in the literature regarding the usefulness of standard waves, such as N1 and P3, in evaluating differences in the levels of earlier processing (“perception”) and lasting processing (“attention”), respectively. Therefore, below we will review the literature of the impact of anesthetics mainly on N1 and P3 (but, occasionally, also on other early and later waves) as indicators for their impact on perception and attention, respectively.

Our goal was to learn from ERP about consciousness while under anesthesia. What do we gain from the fact that ERP might be a combination of such perception-related and attention-related processes? Possibly, in order to address this question, we may need to take a step back and to remember there is a debate concerning the neurophysiological basis of consciousness in general. Indeed, there are scholars who believe that consciousness is too complex and irreducible to a set of neuropsychological processes. According to this approach, one may only explore for electrophysiological correlates with consciousness (e.g., by way of data mining “fishing expeditions”), and it would be futile to try and model the phenomenon of consciousness in neuropsychological terms (Lamme, 2006). Although it is certainly ambitious to offer a neuropsychological model for consciousness in general, we aim, in this work, for a humbler goal: awareness of aversive and nociceptive conditions under anesthesia and during surgery. Such stressing conditions are likely to evoke perception and attention processes. Therefore, in the context of anesthesia, it might be possible to replace the somewhat abstract discussion of neurophysiological markers for consciousness, with the more concrete neuropsychological discussion of specific markers, which seem to be attention related and perception related and which seem to support conscious experience.

Certainly, the precise definition of various neuropsychological processes, such as perception and attention, and their underlying neurophysiological processes might be by itself challenging and requires in-depth discussion (Mesulam, 1998; Posner and Rothbart, 2007). Nevertheless, the literature, which associates between these processes and classical ERP waves, such as P3 with attention and higher cognitive processing and N1 with sensation and perception, is well established and stands at the core of the discussion below.

## The Orderly Impact of Anesthetics on Event-Related Potential and Cognition—Attention Is Affected Before Perception

### The Attention-Related Process Is Affected Firstly

It seems that, for example, P3 (and other attention-related waves) and N1 (and other perception-related waves) are not inhibited evenly by anesthetic drugs (when we refer here to wave inhibition, we include both amplitude reduction and latency increase), in sub-anesthetic doses (namely, doses that do not reach loss of consciousness). There seems to be an order, whereby P3 is more inhibited first, also with sub-anesthetic doses of various drugs, while N1 is significantly less affected. See, for example, the literature regarding propofol (Reinsel et al., 1995), ketamine (Musso et al., 2011), nitrous oxide (Jessop et al., 1991), and midazolam, fentanyl, and thiopental (Veselis et al., 2001). Furthermore, in the sub-anesthetic range, there seems to be an association between the drug dose and P3 wave inhibition (Jessop et al., 1991; Reinsel et al., 1995).

Notably, at the sub-anesthetic range, it is possible to conduct various cognitive evaluations. Thus, it seems that it is also possible to find deterioration in performance of tasks, which evaluate attention directly (Umbricht et al., 2000). However, it is also possible to find global deterioration in multiple other higher cognitive functions—such as executive functions and memory (Veselis et al., 1992; Sarasin et al., 1996). Still, one thing these different neuropsychological functions have in common is that they all involve attention (Chun and Turk-Browne, 2007; Gazzaley and Nobre, 2012). Thus, it seems plausible that, indeed, attention is hindered by sub-anesthetic drugs. In fact, it seems that the deterioration in cognitive performance, under sub-anesthetic dose of the various drugs, is associated with the degree of inhibition to P3 and other attention-related (or higher cognitive processing) waves (Jessop et al., 1991; Reinsel et al., 1995; Umbricht et al., 2000). Thereby, further support is provided to the early impact of the various drugs on cognition and, specifically, on attention and to its manifestation in P3 and other attention-related waves.

### The Perception-Related Process Is Affected Secondly

With increase of anesthetic dose, it is possible to see, with multiple drugs, also a reduction of N1 and other perception-related waves (propofol—Simpson et al., 2002; midazolam—Veselis et al., 2001; nitrous oxide—Jessop et al., 1991). In all of these instances, this reduction is accompanied by further reduction in P3 and other attention-related waves. Behaviorally, this reduction of N1 is often manifested at the time of loss of consciousness (Vaselis et al., 2001; Simpson et al., 2002). In fact, loss of consciousness is generally measured clinically by reduced responsiveness (Alkire et al., 2008). But reduced sensation and perception would result in reduced responsiveness. Thus, it may provide support to the assumption that the anesthetic drug effect of reduced perception manifests with reduction in early waves, such as N1.

Interestingly enough, not all anesthetic drugs result in reduction of N1, even at the level of loss of consciousness. For example, ketamine, at anesthetic dosages, does not seem to reduce N1 (Schwertner et al., 2018). However, there is still reduction of P3 and attention-related waves. Thus, possibly, this reduction of attention-related activity might be sufficient to reduce sensation and perception, as would be suggested below. Furthermore, we suggest below that such a differed reduction of attention-related processes, which may manifest with ketamine, could be sufficient for loss of consciousness but may also bring about psychotic symptoms (hallucinations and delusions; Corlett et al., 2007). In fact, it seems that the onset of psychotic symptoms may be associated with such differed reduction of P3, especially in posterior cortical regions (Musso et al., 2011).

Interestingly, ketamine is considered as a model for schizophrenia (Kocsis et al., 2013). Probably, owing to the pharmacological orientation of psychiatric treatment, leading models of schizophrenia focus on the role of various neuromodulators and neurotransmitters (Egerton and Stone, 2012; Howes and Murray, 2014). However, we, as well as others, suggested a neurophysiology-based top-down mechanism for schizophrenia and especially for its psychotic symptoms (Shahaf, 2016, 2019). According to this mechanism, perception is based not only on bottom-up sensory activation but also on top-down activation from frontal regions, which contributes to the activation of the posterior perceptual regions. Thus, psychotic symptoms in schizophrenia might be the result of reduced frontal activation. However, ketamine-induced reduced attention would also cause reduced frontal activation and thereby reduced top-down activation of the perceptual regions, which will lead to psychotic symptoms. Thus, with certain similarity to the proposed mechanism in schizophrenia, ketamine-induced psychotic symptoms might simply be the result of such differed reduction of attention, frontal activity, and top-down activation of perceptual regions, as opposed to co-reduction of both attention and perception, with other drugs.

This means that even the challenging phenomenon of ketamine-induced psychotic symptoms might merely be the result of differed reduction of attention and might manifest as further reduction in P3 and other related electrophysiological markers for attention. Nevertheless, this further reduction of attention might also form an alternative route to loss of consciousness, in addition to combined reduction of attention and perception (and P3 and N1 and other attention-related and perception-related waves). Indeed, the psychotic symptoms might require some level of activity in the perception regions. Thus, the reduction of this activity beyond a certain level, as manifested, for example, by reduced N1, might reduce the likelihood of psychotic symptoms.

However, as we suggested above, this reduction of perception, which manifests as reduction in N1 and other waves, for the other drugs, might be an important contributor for loss of consciousness. It might be that with ketamine, it is the further reduction of attention and its top-down activation, from prefrontal regions to perception regions, that eventually also hinders perception functionality and causes loss of consciousness (with the possible expense of psychotic symptoms).

Notably, there is an electrophysiological difference between ketamine effect and schizophrenia with regard to N1 (Schwertner et al., 2018). N1 reduction is observed in schizophrenia but is not similarly induced by ketamine. Our model for schizophrenia is focused on the reduction of top-down activation from the amygdala to perception regions (Shahaf, 2016). Thus, owing to the relatively early involvement of the amygdala, it is likely to reduce the activity of early waves, such as N1. On the other hand, the inhibitory effect of ketamine, at the later level of the prefrontal cortex, may impact only the later waves, such as P3. Indeed, such later waves might relate to top-down processing, which involves the prefrontal cortex and its working memory interaction with perception regions (Dong et al., 2015). Thus, it is possible that top-down activations from both the amygdala and the prefrontal cortex might be required for effective activation of posterior perception regions (and avoidance of psychotic symptoms). However, whereas in schizophrenia the deficit may be in the earlier amygdala activation, in drug-induced psychotic symptoms the deficit may be in the later working memory, and, thereby, does not result in reduced N1 and other perception-related waves, which precede it in time. In a sense, this difference, between schizophrenia and drug-induced psychotic symptoms, provides further support to the suggestion that drug-induced P3 changes represent reduced attention and its impact on prefrontal processes.

## Implications

### Theoretical Implications: Neuropsychological Focus on Attention and Perception Instead of on Consciousness

All in all, we suggest in this work to focus on two types of neuropsychological processes, which may have a role in consciousness while under anesthesia—namely, attention-related processes and perception-related processes. Furthermore, we suggest that loss of consciousness will result from their combined reduction or alternatively from a sufficient reduction of attention-related processes *per se* (which will still hinder also perception in a secondary manner). This combined reduction of attention and perception would lead to reduced patient’s responsiveness. Furthermore, we (Shahaf et al., 2016), as well as others, also suggested that the reduction of (mainly) attention-related processes would also lead to amnesia (which is considered to be another component, beyond responsiveness, in the loss of consciousness—Alkire et al., 2008). The interaction between responsiveness to aversive and nociceptive conditions during surgery and their memory thereafter seems to involve also additional processes, which require further research (Bonhomme et al., 2019). Nevertheless, the contribution of perception and attention and their ERP manifestation to memory formation seems well established (Pryor et al., 2010). Thus, all in all, we suggest a common pathway to the loss of consciousness for the various drugs, of variable molecular and cellular impact—namely, reduced attention, which is followed, one way or the other, also by reduced perception.

### Practical Implications: A Possible Route to Develop a Marker for Depth of Anesthesia

Based on the above, it might be possible to develop ERP-based markers for consciousness level under anesthesia, or as we suggest of combined attention level and perception level. However, as we suggested above, the standard ERP waves might be useful at the group level but not reliable at the single sample level, especially when continuous, real-time monitoring is considered. Also, as was presented above, other prevailing methods, such as blind source separation, might also not be sufficiently precise for implemental purposes. Thus, it seems that novel methods of analysis might be required for developing an effective practical ERP monitor, which would be applicable for various anesthetic drugs. Possibly, it would be interesting to explore for this aim a combination of the attention-related and perception-related indices we developed (Shahaf and Pratt, 2013; Shahaf et al., 2015).

**Figure 1:**
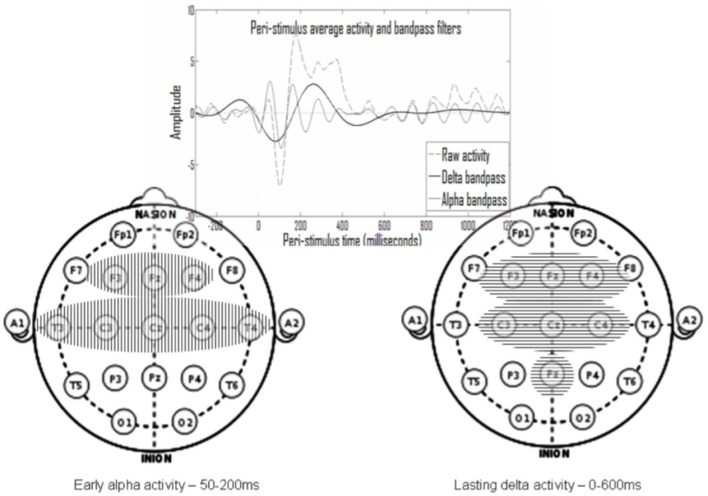
The temporal and spatial spread of the two processes (taken from Shahaf and Pratt, 2013). An oscillatory early alpha activity (mainly between 50 and 200 ms after stimulus) was identified in eight electrodes (left) and an oscillatory prolonged delta activity (mainly between stimulus onset and 600 ms thereafter) was identified in seven electrodes (right). The alpha activity was shown to associate with perception and with standard event-related potential (ERP) waves associated with perception, and the delta activity was shown to associate with attention and with standard ERP waves associated with attention.

As one example of the feasibility for effective decomposition of the multi-channel ERP signals to two types of underlying processes, one of which could be associated with perception and the other associated with attention, see Shahaf et al. (2015). Figure 1, taken from Shahaf and Pratt (2013), shows the end product of such decomposition. The averaged ERP signal is decomposed into oscillations in the delta and alpha frequency bands, which were found to associate with attention- and perception-related processes, respectively. The perception-related alpha oscillations last for ~150 ms, whereas the attention-related delta oscillations last ~600 ms. As could be seen in the figure, both markers are spread spatially, which means that they could be sampled from few electrodes. However, the current manuscript merely aims to present a theoretical framework and to explain the rationale for focusing on attention- and perception-related markers. Therefore, the demonstration of their usefulness in anesthesia is left for other more experimental reports. Furthermore, these very specific attention- and perception-related markers should only be considered as one possible feasible example, and various other such markers could be devised and evaluated.

**Figure 2:**
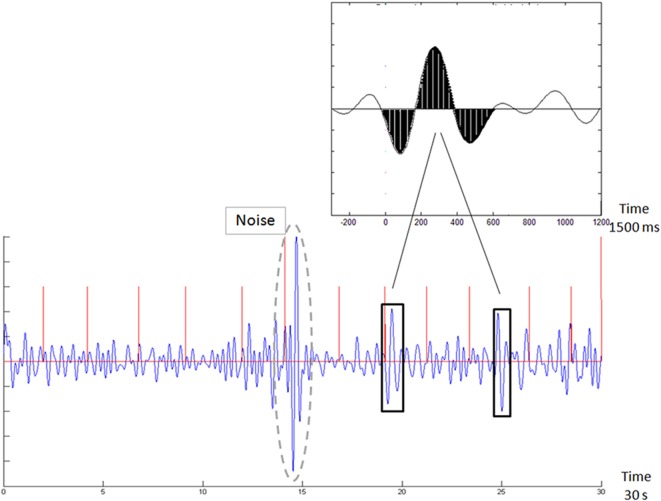
Template matching of the averaged ERP signal in the raw electroencephalogram (EEG) sample (taken from Shahaf et al., 2017). The top inset shows the pattern of the attention-related marker, which is shown in Figure 1. The bottom figure shows the exploration for this pattern in the continuous EEG signal. Vertical red lines mark (auditory oddball) stimulus times. Two matches are marked (crossed the matching threshold).

Furthermore, it would be simpler, in operative terms, if effective markers could be derived in real time also from the raw EEG data, without specific timing to external stimuli, and especially without the need to average multiple and lengthy repetitions of event-related activity. In this context, we, and others, suggested a relation between the ERP signal and the background EEG oscillations throughout the task (Shahaf et al., 2015). We followed in other clinical conditions the approach of task-related EEG analysis (Loo et al., 2009), and we obtained effective results, at least as far as attention monitoring is considered (citealpB50,B48,B49). In these studies, we used a template matching technique to explore the raw EEG data for the presence of the patterns, which were first identified in the averaged ERP. Figure 2, taken from Shahaf et al. (2017), demonstrates how the attention-related template, presented in Figure 1, is identified in the task-related data without averaging. The algorithmic description of this matching could be found in Shahaf et al. (2017) and in other texts cited above. However, it should be remembered again that this is only one feasible example, which is given for illustrative purposes only, and the focus of the current manuscript is to present the theoretical framework, for which other embodiments might also be devised.

Furthermore, it might be that such background oscillatory markers could be analyzed even from raw EEG, without the context of a specific task. In fact, we obtained some interesting results also with “task-less” raw EEG data in various clinical contexts (Bartur et al., 2017, 2019; Isserles et al., 2018; Shahaf et al., 2018b). Furthermore, some initial results were also obtained during the use of etomidate of localized cortical anesthesia during WADA test (Shahaf et al., 2016).

We will leave for future, more experimental works, the suggestion of specific markers for consciousness while under anesthesia (or, in our terms, of combined attention and perception). All in all, the purpose of the current work was to present the theoretical framework.

## Author Contributions

DB, GH, and GS wrote the main manuscript text and approved it.

## Conflict of Interest

DB and GS are employed by NeuroIndex Ltd., which develops EEG markers for anesthesia. However, the article presented here is a theory and hypothesis article based on a review of the general literature, without any presentation of NeuroIndex technology or results.

The remaining author declares that the research was conducted in the absence of any commercial or financial relationships that could be construed as a potential conflict of interest.
